# Virtually Unexpected: No Role for Expectancy Violation in Virtual Reality Exposure for Public Speaking Anxiety

**DOI:** 10.3389/fpsyg.2019.02849

**Published:** 2019-12-17

**Authors:** Sara Scheveneels, Yannick Boddez, Tom Van Daele, Dirk Hermans

**Affiliations:** ^1^Centre for the Psychology of Learning and Experimental Psychopathology, KU Leuven, Leuven, Belgium; ^2^Department of Clinical Psychology and Experimental Psychopathology, University of Groningen, Groningen, Netherlands; ^3^Expertise Unit Psychology, Technology & Society, Thomas More University of Applied Sciences, Antwerp, Belgium

**Keywords:** virtual reality, exposure therapy, expectancy violation, anxiety, retrospective reasoning

## Abstract

In the current study, we examined the role of expectancy violation and retrospective reasoning about the absence of feared outcomes in virtual reality exposure therapy (VRET). Participants fearful of public speaking were asked to give speeches in virtual reality. We asked each participant individually to report their expectancies about feared outcomes in public speaking situations and which of these could be tested in VRET. Each of the expectancies was categorized as being related to: (1) participants’ own reactions, (2) (overt) reactions of the audience, or (3) (covert) negative evaluation. We examined whether the proportion of testable expectancies could predict treatment outcome and which type of expectancies were evaluated as being more testable in VRET. Additionally, we experimentally manipulated retrospective reasoning about whether or not expectancies related to the overt reactions of the audience could be violated by providing verbal information after VRET about whether or not the virtual audience was interactive. A reduction in public speaking anxiety was observed from pre- to post-VRET. Treatment effects were, however, not predicted by the individually reported proportions of testable expectancies. Participants evaluated expectancies about their own reactions as being more testable in VRET compared to expectancies about reactions of the audience or about being negatively evaluated. In addition, we did not find evidence that the experimental manipulation regarding whether or not the audience was interactive influenced treatment effects. In conclusion, the results of the current study suggest that the effects of VRET are not univocally explained by the mechanism of expectancy violation.

## Introduction

Exposure involves the repeated confrontation with fear-evoking stimuli and is a key component in the treatment of anxiety (e.g., Wolitzky-Taylor et al., 2008; Öst et al., 2015). Typically, this confrontation takes place in real life. For example, a client with fear of flying would be encouraged to take a flight. However, such exposure *in vivo* could be demanding for both the client and the therapist. Immediate and direct confrontation with the phobic situation is sometimes considered as too threatening by the client, leading to low treatment acceptance and dropout (Choy et al., 2007). For the therapist, organizing an *in vivo* exposure session can be time-consuming, for example when one has to gather an audience for an exercise in public speaking anxiety.

Virtual reality exposure therapy (VRET) can overcome some of these challenges. In VRET the phobic situation is generated by a computer using VR technology rather than by the natural environment (Stever, 1992). This can provide easy access to a wide range of exposure exercises without leaving the therapist office. Moreover, it has been demonstrated that VRET is associated with higher treatment acceptability in phobic individuals (Garcia-Palacios et al., 2007, but see Opriş et al., 2012). Besides these advantages, empirical evidence confirms the effectiveness of VRET for anxiety reduction. Meta-analyses support that VRET is superior to waitlist control and that VRET-gains generalize to real life (Powers and Emmelkamp, 2008; Morina et al., 2015). Moreover, VRET has found to be equally effective as exposure *in vivo* on the short- and long-term (Powers and Emmelkamp, 2008)^1^.

Inhibitory Learning Theory (ILT) explains the success of exposure by the formation of an inhibitory association which counteracts the original (excitatory) fear association (e.g., between “taking a plane” and “crashing”) that drives fear responding (Craske et al., 2008, 2014). Since it is assumed that the relative strength of the inhibitory and excitatory association determines fear responding, ILT postulates that inhibitory learning should be maximized during exposure. In doing so, it is assumed that the concept of expectancy violation plays a central role. In particular, exposure should be directed at providing a strong mismatch between the client’s expectancies for the likelihood of an aversive outcome and the actual outcome. The more the expectancy can be violated during exposure, the stronger the inhibitory learning (Deacon et al., 2013; Craske et al., 2014).

Certain types of expectancies are, however, difficult to test and violate in VRET. In particular, VRET might be less appropriate to test and violate particular expectancies compared to exposure *in vivo* because certain outcomes cannot occur in VR. For example, in VRET for fear of flying, feared outcomes like an actual injury or death because of a plane crash cannot occur. In light of this, the success of VRET seems quite paradoxical. Other types of feared outcomes can, however, be tested in VRET. This might be particularly true for feared outcomes toward one’s own reactions. In the fear of flying example, a plane crash cannot occur in VRET, but expected outcomes related to own reactions such as having a panic attack can. In line with the theoretical assumptions of ILT this would imply that those clients whose fear of flying is primarily driven by the expectancy of a plane crash might benefit less from VRET compared to those clients whose fear is driven by the expectancy of having a panic attack.

Nevertheless, a sense of presence (i.e., the connection an individual feels with the VR environment) can explain why an individual experiences VR “as if it is real” and might come to expect certain outcomes to occur even though it is actually impossible (Price and Anderson, 2007; Price et al., 2011). However, even then there is the risk that the individual retrospectively reasons that certain aversive outcomes could not occur because treatment took place in VR. Experimental research has shown that such retrospective reasoning about the absence of the aversive outcome can cause a return of fear responding. Using a differential fear conditioning procedure, Raes et al. (2011) first paired one stimulus with an electric shock. In subsequent extinction, the stimulus was no longer paired with the shock, as an analog for exposure therapy. After extinction, the experimental group received information that due to a technical failure the shock could not occur during extinction. These participants showed higher fear responding in a subsequent test phase compared to a control group that did not receive this information. Translating these results to VRET, retrospective reasoning that the feared outcome could not have occurred might interfere with the effects of VRET.

The present study examined the role of expectancy violation and retrospective reasoning about the absence of feared outcomes in VRET. We used a clinical analog sample of participants highly fearful of public speaking. First, we investigated whether the amount of expectancies that participants rated as being testable in VRET could predict the decrease in public speaking anxiety after VRET. To this end, before VRET, each participant was asked about his or her expectancies in public speaking situations. After VRET, we examined which of their expectancies participants evaluated as being testable in VRET. We calculated the proportion of testable expectancies for each participant and tested whether this proportion predicted the treatment effects of VRET. In line with the role of expectancy violation in ILT, it was predicted that participants who evaluated their expectancies about feared outcomes as being more testable in VRET would benefit more from it. Moreover, each of the expectancies was categorized as being related to (1) participants’ own reactions (e.g., I will stutter), (2) (overt) reactions of the audience (e.g., People will ask difficult questions), or (3) (covert) negative evaluation (e.g., They will think that I am incompetent). It was hypothesized that expectancies with regard to own reactions (e.g., going out of my mind) would be evaluated as being more testable in VRET than expectancies related to reactions of the audience or being negatively evaluated. Moreover, it was predicted that participants who reported a higher proportion of expectancies related to their own reactions relative to other expectancies would benefit more from VRET. Second, in an experimental part of this study, we translated the fear conditioning study of Raes et al. (2011) to a VRET procedure. After the two VRET-sessions, participants in the “interactive condition” were instructed that the virtual audience could have reacted on their presentations. Participants in the “non-interactive condition” received the information that such interaction was not possible. As such participants in this group were encouraged to retrospectively reason that feared expectancies about the overt reactions of the audience could not have been tested and violated during VRET. In line with the findings of Raes et al. (2011), we hypothesized higher post-treatment fear responding in the non-interactive condition compared to the interactive condition. Finally, as a proof of concept, this study also enabled us to examine whether a decrease in public speaking anxiety was observed after VRET using low-cost VR-technology (i.e., 360° movie clips).

## Materials and Methods

### Participants

Forty-three participants (38 females), all fearful of public speaking, participated in this study (mean age = 22.72; *SD* = 4.64). Participants were screened relying on a two-item questionnaire that was used in previous studies (Tsao and Craske, 2000; Culver et al., 2012; Niles et al., 2015). The items assessed (1) how anxious they would feel when giving a formal speech in front of a live audience and (2) the extent to which they would avoid taking a class that required giving an oral presentation. In line with these previous studies, respondents scoring a 6 or higher on anxiety and a 5 or higher on avoidance (on a scale ranging from 0 = none/never to 8 = extremely/always) were recruited for participation. Two hundred eighty respondents completed the two screening questions, 102 of them qualified for participation and were contacted by the experimenters. Twenty-two participants were randomly allocated to the interactive condition and 21 participants to the non-interactive condition. Participants received course credit or financial compensation. The study was approved by the ethical committee of the Faculty of Psychology and Educational Sciences of KU Leuven.

### Measures

#### Self-Report Questionnaires

##### Personal report of confidence as a speaker (PRCS; Paul, 1966)

The PRCS consists of 30 true/false items to assess participants’ confidence as a public speaker. Previous research supports the internal consistency (α = 0.91) and validity of the PRCS showing correlations between 0.52 and 0.97 with other measures of social anxiety (Klorman et al., 1974; Daly, 1978). Moreover, the PRCS has shown to be sensitive to treatment (Lawm et al., 1994).

##### Self-statements during public speaking scale (SSPS; Hofmann and DiBartolo, 2000)

The SSPS consists of 10 items measuring thoughts and feelings during public speaking. Items are scored on a scale between 0 (= totally disagree) and 5 (= totally agree). Five items measure positive statements (SSPS-P) and five items negative statements (SSPS-N). Internal consistency has shown to be high for the SSPS-P (α = 0.80) and SSPS-N (α = 0.86). Test-retest reliability was acceptable for the SSPS-P (*r* = 0.78) as well as the SSPS-N (*r* = 0.80). In addition, the SSPS-N, but not the SSPS-P, has found to be sensitive to treatment (Hofmann and DiBartolo, 2000).

##### List of expectancies

Based on the existing literature and available questionnaires, we developed a list of 50 expectancies commonly reported by individuals with speaking anxiety (Supplementary Material A). This list contains 25 expectancies about individuals’ own reactions (e.g., I will stutter), 14 expectancies related to being negatively evaluated by others (e.g., They will think that I am incompetent) and 11 expectancies about the reactions of the audience (e.g., People will ask difficult questions). Participants rated the expectancies on a yes/no answer format. Before the VRET we asked participants to indicate for each expectancy whether or not it is applicable to them in public speaking situations. We calculated the internal consistency for the entire scale (α = 0.86) as well as for the subtypes (own reactions: α = 0.75; reactions of the audience α = 0.86; negative evaluation: α = 0.79). After the VRET participants were asked to indicate for each of the expectancies whether or not it was possible to test the expectancy in the VRET exercises (irrespective of whether or not it happened). For each participant, the proportion of testable expectancies was computed by taking the overlap between expectancies the participant reported in public speaking situations (at pre-assessment) and which expectancies could be tested in VRET. This number was then divided by the total number of expectancies reported by the participant in public speaking situations (at pre-assessment). The proportion of testable expectancies was computed for the total set of expectancies, as well as for each subtype (self, negative evaluation, audience).

#### Behavioral Avoidance Test

In the BAT, participants gave a speech while standing in front of a live audience of two females and one male. Participants were instructed to speak for as long as they could, up to 2 min. Subsequently, participants were told that they had successfully completed the speech and that they had the possibility to continue with their speech for a maximum of another 2 min. It was emphasized that this was not mandatory and that it was entirely the choice of the participant whether or not to continue. The duration of the speeches was registered as a behavioral index. At each of the two assessment sessions, a (at the time in Belgium controversial) topic for the BAT-speech was randomly picked by the participant out of two possibilities: (1) “Is it desirable to put a ban on headscarves?” and (2) “Should there be limitations on the earnings of managers, sportsmen, etc.?”. Once picked, a topic was removed from the pool. Hence all participants in the end presented about the same topics, but whether a particular topic was picked at pre- or post-assessment could vary. Participants were not allowed to prepare their BAT-speeches.

During the BAT, we monitored Subjective Units of Distress (SUDS; Wolpe, 1973). Participants were instructed to give a rating between 0 and 100, where 0 = no fear, 25 = mild fear, 50 = moderate fear, 75 = severe fear, and 100 = very severe fear. SUDS ratings were asked just before the start of the speech, after 1 min and after 2 min (just before participants ended their speech). For each BAT, an average across all SUDS ratings was calculated.

A Polar RS800CX (Polar Electro Oy, Kempele, Finland) was used to measure heart rate (HR; beats per minute) during the BAT. Participants wore a wristband that receives data from a chest-strap also worn by the participant. Inter-beat intervals were sampled at a frequency of 1000 Hz. Empirical evidence supports the validity and reliability of this type of HR monitors in research (Weippert et al., 2010; but see Quintana et al., 2012; Wallén et al., 2012). A 5-min baseline-HR was measured at pre- and post-assessment while participants were seated and before they received instructions about the BAT. ARTiiFACT software (Kaufmann et al., 2011) was used for automated artifact detection, for the handling of missing data and deletion of artifacts. After processing, a difference score was computed between the average HR measured during the BAT and the average baseline-HR.

### Procedure

The study consisted of four sessions that took place on consecutive days for any participant enrolling in the study: pre-assessment, two VRET-sessions and post-assessment.

At the start of pre-assessment, participants signed the informed consent. Next, baseline-HR was recorded while participants were left alone in the room and were instructed to sit quietly. Subsequently, participants received instructions about the BAT. After the BAT, participants completed the PRCS, SSPS and list with expectancies.

Two VRET-sessions were scheduled on the next 2 days. Each of these contained two exposure exercises in which participants were asked to give a speech about a (controversial) topic in front of a virtual audience. There were four different topics: (1) “In which circumstances is abortion justified?”, (2) “Are we spending too much time on social media?”, (3) “Is it desirable to put restrictions on immigration?”, (4) “How should we deal with long-term unemployment?”. All participants presented about every topic, but the order was picked randomly by each participant. During these exercises, participants wore a Samsung Gear VR headset with a Samsung S7 smartphone inserted on which the 360° movie clips were displayed. These movie clips, recorded using a Samsung Gear 360°camera, contained recordings of four audiences varying in composition, duration and context. In the first exercise, an audience of four people was seated in an office. The second exercise took place in a meeting room in front of an audience of 12 people. In the third exercise participants gave their speech in a class room in front of an audience of 20 people. In the fourth exercise an audience of approximately 150 people was seated in an auditorium. Since the movie clips were pre-recorded, they were exactly the same for all participants and audiences were not interactive. Participants had 5 min preparation time for each speech. They did not know the duration of the speech or composition of the audience in advance. At the start of the first VRET-session, participants were presented with an empty room to get accustomed to the VR environment and device. At the end of the second VRET-session, participants completed the list of expectancies.

Post-assessment started with the experimental manipulation. Participants received an information sheet framed as a non-disclosure agreement concerning the technology used in the study (Supplementary Material B). They were instructed to carefully read the information and sign it. The non-interactive condition was informed that the used technology did not allow for an interactive audience and that therefore the audience could not react on their speeches. It was added that if they said something odd or stupid, they would not have noticed this in the reactions of the audience. In the interactive condition it was stated that we used new technology that allowed the audience to react on the participants’ speeches. Here it was added that if they said something odd or stupid, they could have noticed this in the reactions of the audience. The information was repeated orally by the experimenter. Subsequently, baseline-HR was recorded. After this, participants picked a topic and completed the BAT, followed by the PRCS, SSPS and list of expectancies. Finally, we asked participants to indicate how credible it was that the audience was interactive/not interactive. They could choose from four possibilities: (1) Not credible at all; (2) Not very credible; (3) Somewhat credible; and (4) Very credible.

One week after post-assessment, participants completed the PRCS and SSPS online.

## Results

### Does the Proportion of Testable Expectancies Predict Treatment Effects of VRET?

Using a sum score of the different types of expectancies, we tested whether the individually calculated proportion of testable expectancies (measured before the experimental manipulation) predicted treatment effects. Treatment effects of VRET were represented by a difference score between pre- and post-assessment outcome measures. For each outcome measure (BAT-SUDS, BAT-HR, PRCS, SSPS-P, SSPS-N) we conducted a linear regression analysis with proportion of testable expectancies as the predictor variable and the pre-post difference score as the outcome variable. We used the proportion of testable expectancies measured before the experimental manipulation to avoid distortion by the experimental manipulation. Treatment effects, however, were measured after the experimental manipulation. To explore whether the relation between the proportion of testable expectancies and treatment effects is moderated by experimental condition, we added condition as a moderator in the regression analyses. Since we performed five regression analyses, we applied Bonferroni correction and levels for significance were set at 0.01. Results of the analyses are displayed in Table 1. None of the results was significant at the 0.01 level. Only for BAT-SUDS, the proportion of testable expectancies predicted treatment effects (*p* = 0.05). However, the relation is opposite to what we expected: the higher the proportion of testable expectancies, the lower the pre-post difference in BAT-SUDS. This effect was not moderated by condition. In addition, the moderation effect approached the 0.05 criterion for significance in the BAT-HR (*p* = 0.07) and PRCS (*p* = 0.06). However, inspection of the individual correlations did not reveal a clear pattern. For the BAT-HR, correlations are in the predicted direction in the interactive condition (*r* = 0.27), but not in the predicted direction in the non-interactive condition (*r* = −0.31). For the PRCS, correlations are in the predicted direction in the non-interactive condition (*r* = 0.20), but not in the predicted direction in the interactive condition (*r* = −0.40). For explorative reasons, we also calculated partial correlations between the proportion of testable expectancies and treatment effects, controlled for condition (Table 2). These results are in line with the results of the regression analyses.

**TABLE 1:** Multiple linear regression analyses predicting treatment effects from proportion of testable expectancies, condition and proportion of testable expectancies × condition.

	***B***	***SE B***	***B***	***t*-Value**	***p*-Value**
**BAT-SUDS**					
Intercept	18.34	7.58		*t*(3) = -2.42	*p* = 0.02
Expectancies	–16.57	8.06	–0.31	*t*(3) = -2.06	*p* = 0.05
Condition	4.01	3.99	0.15	*t*(3) = 1.00	*p* = 0.32
Expectancies × condition	–3.11	2.06	–0.23	*t*(3) = -1.51	*p* = 0.14
**BAT-HR**					
Intercept	14.89	6.00		*t*(3) = 2.48	*p* = 0.02
Expectancies	–1.86	6.38	–0.04	*t*(3) = -0.29	*p* = 0.77
Condition	–3.26	3.16	–0.16	*t*(3) = -1.03	*p* = 0.31
Expectancies × condition	3.03	1.63	0.28	*t*(3) = 1.86	*p* = 0.07
**PRCS**					
Intercept	6.15	3.16		*t*(3) = 1.95	*p* = 0.06
Expectancies	–2.38	3.37	–0.11	*t*(3) = -0.71	*p* = 0.49
Condition	0.41	1.67	0.04	*t*(3) = 0.25	*p* = 0.81
Expectancies × condition	–1.66	0.86	–0.30	*t*(3) = -1.93	*p* = 0.06
**SSPS-P**					
Intercept	–4.88	1.85		*t*(3) = -2.64	*p* = 0.02
Expectancies	–0.67	1.97	–0.05	*t*(3) = -0.34	*p* = 0.74
Condition	1.55	0.98	0.25	*t*(3) = 1.59	*p* = 0.12
Expectancies × condition	0.32	0.50	0.10	*t*(3) = 0.63	*p* = 0.53
**SSPS-N**					
Intercept	2.82	1.48		*t*(3) = 1.91	*p* = 0.06
Expectancies	0.16	0.78	0.03	*t*(3) = 0.20	*p* = 0.84
Condition	–2.22	1.58	–0.22	*t*(3) = -1.41	*p* = 0.17
Expectancies × condition	–0.54	0.40	–0.21	*t*(3) = -1.34	*p* = 0.19

**TABLE 2:** Partial correlations between proportion of testable expectancies and treatment effects (pre-post).

	**PRCS**	**BAT-SUDS**	**BAT-HR**	**SSPS-P**	**SSPS-N**
Proportion of testable	−0.07	−0.28	−0.08	−0.07	−0.19
expectancies	(*p* = *0.66*)	(*p* = *0.07*)	(*p* = *0.60*)	(*p* = *0.67*)	(*p* = *0.23*)

### Are Expectancies About Individuals’ Own Reactions Better Testable in VRET?

**FIGURE 1:**
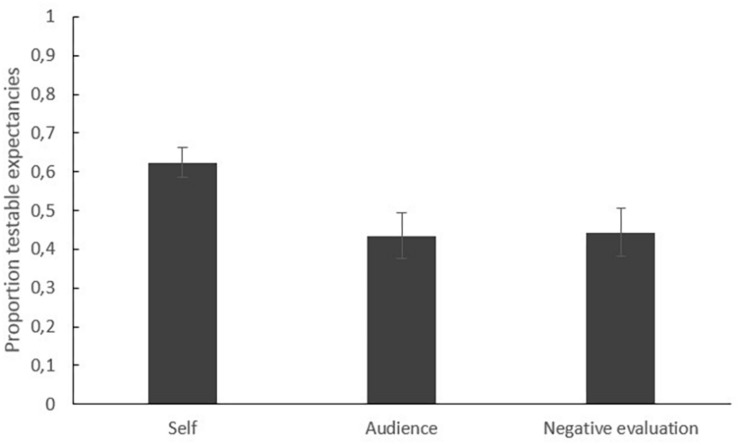
Mean proportions of testable expectancies per type of expectancy (self, audience, negative evaluation) measured after VRET (before the experimental manipulation).

Figure 1 displays the mean proportions of testable expectancies per type of expectancy as measured after VRET (before the experimental manipulation). We tested whether VRET is more eligible to test expectancies about own reactions compared to expectancies about the overt reactions of the audience or being negatively evaluated. This was confirmed by a significant main effect of type of expectancy in a repeated measures analyses of variance (rmANOVAs) on the proportion of testable expectancies with type of expectancy as within-subjects factor, *F*(2, 84) = 7.02, *p* = 0.002, η*^2^p* = 0.14.

### Do Individuals With Higher Proportions of Expectancies About Their Own Reactions Benefit More From VRET?

Given that expectancies about one’s own reactions are better testable in VRET, it was predicted that individuals with relatively higher proportions of such expectancies benefit more from VRET. To answer this question, we calculated a new variable representing the proportion of expectancies about one’s own reactions. This was done by dividing for each participant the number of expectancies about one’s own reactions by the total number of expectancies at pre-assessment. Linear regression analyses were conducted for each outcome measure with proportion of expectancies related to own reactions as the predictor variable and a difference in outcome between pre- and post-assessment as the outcome variable. For explorative purposes, condition was added as a moderator. We applied Bonferroni correction and levels of significance were set at 0.01. Results are displayed in Table 3. Only for the PRCS, the proportion of expectancies about own reactions significantly predicted treatment effects (*p* = 0.007). However, the direction of this relation was opposite to what we predicted: higher proportions of expectancies about own reactions were related to worse treatment effects. This relation was not moderated by condition.

**TABLE 3:** Multiple linear regression analyses predicting treatment effects from proportion of expectancies about own reactions at pre-assessment, condition and proportion of expectancies about own reactions × condition.

	***B***	***SE B***	***B***	***t*-Value**	***p*-Value**
**BAT-SUDS**					
Intercept	19.25	12.44		*t*(3) = 1.55	*p* = 0.13
Expectancies	–17.13	19.94	–0.14	*t*(3) = -0.86	*p* = 0.40
Condition	2.67	4.31	0.10	*t*(3) = 0.62	*p* = 0.54
Expectancies × condition	1.76	2.23	0.13	*t*(3) = 0.79	*p* = 0.44
**BAT-HR**					
Intercept	10.54	9.72		*t*(3) = 1.08	*p* = 0.29
Expectancies	7.45	15.59	0.08	*t*(3) = 0.48	*p* = 0.64
Condition	–2.97	3.37	–0.14	*t*(3) = -0.88	*p* = 0.38
Expectancies × condition	1.43	1.74	0.13	*t*(3) = 0.82	*p* = 0.42
**PRCS**					
Intercept	16.19	4.71		*t*(3) = 3.44	*p* < 0.001
Expectancies	–21.32	7.55	–0.43	*t*(3) = -2.83	*p* = 0.007
Condition	–0.74	1.63	–0.07	*t*(3) = -0.45	*p* = 0.65
Expectancies × condition	0.12	0.84	0.02	*t*(3) = 0.14	*p* = 0.89
**SSPS-P**					
Intercept	–4.60	2.91		*t*(3) = -1.58	*p* = 0.12
Expectancies	–1.08	4.67	–0.04	*t*(3) = -0.23	*p* = 0.82
Condition	1.47	1.01	0.23	*t*(3) = 1.46	*p* = 0.15
Expectancies × condition	0.08	0.52	0.03	*t*(3) = 0.16	*p* = 0.87
**SSPS-N**					
Intercept	3.01	2.40		*t*(3) = 1.26	*p* = 0.22
Expectancies	–2.47	3.84	–0.11	*t*(3) = -0.64	*p* = 0.52
Condition	–0.03	0.83	–0.01	*t*(3) = -0.03	*p* = 0.97
Expectancies × condition	0.15	0.43	0.06	*t*(3) = 0.35	*p* = 0.73

### Is There an Effect of the Experimental Manipulation on Treatment Effects of VRET?

Three participants in the non-interactive condition and four participants in the interactive condition rated the instruction about whether or not the audience was interactive as “not very credible.” All other participants indicated that they found the instruction “somewhat credible” or “very credible.” Statistical analyses after exclusion of the participants who rated the instruction as ‘not very credible’ (*N* = 36) resulted in the same conclusions as analyses including the entire sample (*N* = 43), which are reported here.

Repeated measures analyses of variance (rmANOVAs) with condition (non-interactive, interactive) as between-subjects factor and time (pre-assessment, post-assessment) as within-subjects factor were conducted to test whether the experimental instruction had an effect on the decrease in public speaking anxiety after VRET. We applied Bonferroni correction and levels for significance were set at 0.01.

#### Manipulation Check

As a manipulation check, we compared the proportion of testable expectancies about the reactions of the audience before (after the last VRET-exercise) and after the experimental manipulation between the interactive condition (VR: *M* = 0.53; *SD* = 0.37; post: *M* = 0.57; *SD* = 0.37) and the non-interactive condition (VR: *M* = 0.33; *SD* = 0.39; post: *M* = 0.36; *SD* = 0.37). We expected a decrease in the proportion of testable expectancies about the reactions of the audience in the non-interactive condition, but not in the interactive condition. This was expected because participants in the non-interactive condition were informed that the audience could not react to their speeches. This was, however, not confirmed by a 2 (time: VR, post) × 2 (condition: interactive, non-interactive) rmANOVA. No significant time × condition interaction was found, *F*(1, 41) = 0.01, *p* = 0.98, η*^2^p* = 0.00. This result suggests that participants’ evaluation of which expectancies about the reactions of the audience could be tested during VRET was not influenced by the information that the audience could react to their speeches or not. Nevertheless, we report the analyses below because they test whether there was a decrease in public speaking anxiety after VRET. The effects of the experimental manipulation, however, require careful interpretation given the results of this manipulation check.

Since the experimental manipulation specifically targeted the proportion of testable expectancies with regard to the overt reactions of the audience, we decided beforehand that we would only test the effect of the manipulation on this type of expectancies. For explorative reasons, we also looked at the interactions in the other types of expectancies. A rmANOVA showed no significant time × condition interaction for the proportion of testable expectancies regarding own reactions, *F*(1, 41) = 1.24, *p* = 0.27, η*^2^p* = 0.03. For the proportion of testable expectancies about being negatively evaluated the time × condition interaction was also not significant, *F*(1, 41) = 1.48, *p* = 0.23, η*^2^p* = 0.04.

#### BAT

**FIGURE 2:**
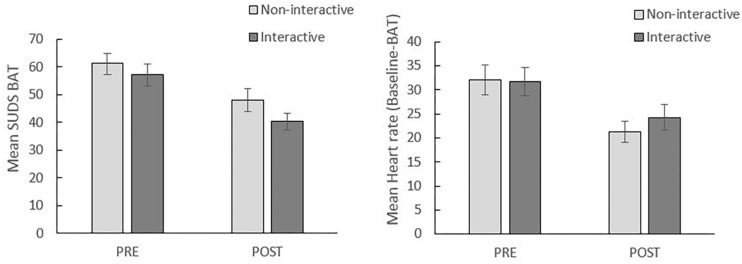
Mean SUDS during BAT (left) and heart rate during BAT (right) per condition at pre-assessment (PRE) and post-assessment (POST). Error bars represent standard errors.

Figure 2 displays the mean SUDS (left panel) and the mean HR (right panel) during BAT for the interactive and non-interactive condition at pre- and post-assessment. The duration of the speeches was not included in the analyses since all participants completed the 2-min BAT speech and only five participants were willing to continue with their speech after the prescribed 2 min (preventing reliable analysis).

##### BAT-SUDS

A 2 (time: pre, post) × 2 (condition: interactive, non-interactive) rmANOVA reveals a significant main effect of time, *F*(1, 41) = 52.25, *p* < 0.001, η*^2^p* = 0.56. This suggests that there was a reduction in BAT-SUDS from pre- to post-VRET (see Figure 2). However, no significant time × condition interaction was found, *F*(1, 41) = 0.74, *p* = 0.40, η*^2^p* = 0.02, indicating that the effect of VRET on SUDS did not differ between the interactive and non-interactive condition.

##### BAT-HR

A significant main effect of time was found in a 2 (Time: pre, post) × 2 (Condition: interactive, non-interactive) rmANOVA, *F*(1, 41) = 32.15, *p* < 0.001, η*^2^p* = 0.44, indicating a decrease in HR from pre- to post-assessment. This decrease did not differ between the interactive and non-interactive condition, given a non-significant Time × Condition interaction, *F*(1, 41) = 1.06, *p* = 0.31, η*^2^p* = 0.03.

#### Self-Report Questionnaires

##### PRCS

**FIGURE 3:**
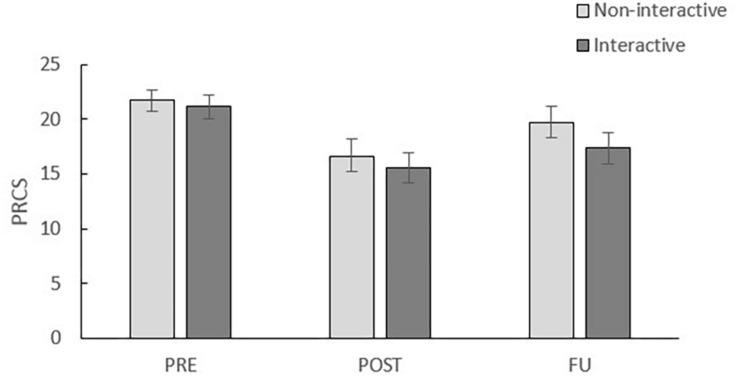
Mean PRCS scores per condition of pre-assessment (PRE), post-assessment (POST) and follow-up (FU). Error bars represent standard errors.

Figure 3 displays the mean PRCS scores per condition at pre-assessment, post-assessment and follow-up. A 2 (time: pre, post) × 2 (condition: interactive, non-interactive) rmANOVA reveals a significant main effect of time, *F*(1, 41) = 39.77, *p* < 0.001, η*^2^p* = 0.49, but no significant time × condition interaction, *F*(1, 41) = 0.04, *p* = 0.84, η*^2^p* = 0.01. These results demonstrate a reduction from pre- to post-VRET in this outcome measure with, however, no differences between both conditions. Although Figure 2 suggests an increase in PRCS score from post-assessment to follow-up, a main effect of time in a 2 (time: pre, FU) × 2 (condition: interactive, non-interactive) rmANOVA demonstrated that treatment effects were (partially) maintained at 1-week follow-up, *F*(1, 41) = 15.83, *p* < 0.001, η*^2^p* = 0.28. No time × condition interaction was found, *F*(1, 41) = 1.34, *p* = 0.25, η*^2^p* = 0.03.

##### SSPS-P and SSPS-N

**FIGURE 4:**
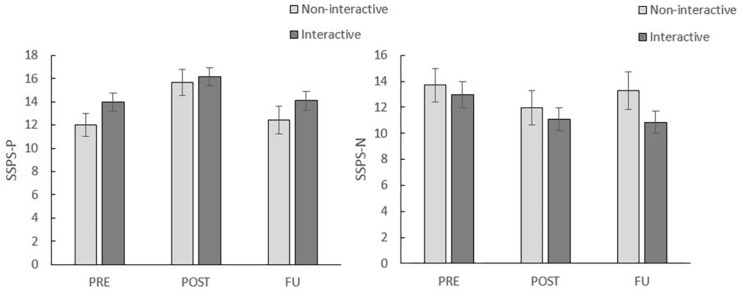
Mean SSPS-P scores (left) and SSPS-N scores (right) per condition at pre-assessment (PRE), post-assessment (POST) and follow-up (FU). Error bars represent standard errors.

Figure 4 displays the mean SSPS-P (left panel) and SSPS-N (right panel) scores per condition at pre-assessment, post-assessment and follow-up. For the SSPS-P a significant main effect of time, *F*(1, 41) = 36.79, *p* < 0.001, η*^2^p* = 0.47, but no significant time × condition interaction, *F*(1, 41) = 2.56, *p* = 0.12, η*^2^p* = 0.06, was observed in a 2 (time: pre, post) × 2 (condition: interactive, non-interactive) rmANOVA. Hence, the effects of VRET were confirmed, with again no effect of the experimental manipulation. Notably, the effects of VRET on the SSPS-P were not maintained at 1-week follow-up: no significant main effect of time in a 2 (time: pre, FU) × 2 (condition: interactive, non-interactive) rmANOVA *F*(1, 41) = 0.23, *p* = 0.64, η*^2^p* = 0.01 was found. The time × condition interaction was not significant, *F*(1, 41) = 0.10, *p* = 0.76, η*^2^p* = 0.01.

Similar results were found for the SSPS-N, with a main effect of time, *F*(1, 41) = 36.79, *p* < 0.001, η*^2^p* = 0.47, and no time × condition interaction, *F*(1, 41) = 2.56, *p* = 0.12, η*^2^p* = 0.06, in a 2 (time: pre, post) × 2 (condition: interactive, non-interactive) rmANOVA. Here, however, effects were maintained at follow-up as demonstrated by a significant main effect of time in a 2 (time: pre, FU) × 2 (condition: interactive, non-interactive) rmANOVA, *F*(1, 41) = 6.55, *p* = 0.01, η*^2^p* = 0.14. The time × condition interaction was not significant, *F*(1, 41) = 2.85, *p* = 0.10, η*^2^p* = 0.07.

## Discussion

The current study investigated the role of expectancy violation and retrospective reasoning about the absence of aversive outcomes in VRET for public speaking anxiety. First, we tested whether the proportion of testable expectancies calculated for each participant individually predicted reductions in public speaking anxiety after VRET. Participants were asked to indicate which expectancies they have in public speaking situations and which expectancies they could test in the VRET. We predicted that participants that were able to test a lot of their expectancies about feared outcomes during VRET would benefit more from it. In addition, it was hypothesized that expectancies about individuals’ own reactions could better be tested in VRET than expectancies about the overt reactions of the audience or about being negatively evaluated and that participants reporting a higher proportion of expectancies related to their own reactions relative to the other types of expectancies would benefit more from VRET. Second, we tested whether retrospective reasoning about the absence of the feared outcome influenced treatment effects. After VRET, we provided participants with information stating that the audience could interact (interactive condition) or could not interact (non-interactive condition). In line with a previous fear conditioning study by Raes et al. (2011), it was predicted that the non-interactive group would benefit less from VRET due to retrospective reasoning that the feared outcomes related to the reactions of the audience could not have occurred. Finally, as a proof of concept, we tested whether VRET using low-cost technology (i.e., 360° movie clips) resulted in a decrease in public speaking anxiety.

The results of this study confirm a reduction in public speaking anxiety from pre- to post-VRET in all outcome measures. We did not find evidence that participants who evaluated their feared expectancies as being more testable in VRET showed a higher decrease in public speaking anxiety after VRET. As hypothesized, expectancies about individuals’ own reactions could better be tested in VRET compared to expectancies about the reactions of the audience and negative evaluation. However, the hypothesis that participants with higher proportions of expectancies about their own reactions would benefit more from VRET was not confirmed. Two remarks can be made with regard to these correlational findings. First, it is possible that participants were not accurate in identifying their expectancies and in evaluating whether these expectancies could be tested during VRET. This could explain why we did not find correlations between (testable) expectancies and treatment effects. However, ILT assumes that clients are aware of and can report their expectancies, which is a necessary requirement for designing an exposure session in which these expectancies can be maximally violated. For example, before an exposure exercise the client is asked about what he expects to happen and afterward the exercise is evaluated with respect to whether the expected outcome occurred (Craske et al., 2014; Weisman and Rodebaugh, 2018). Second, it is possible that participants report certain expectancies that could be tested in VRET, but not all of these might be crucial in their public speaking anxiety. For example, a participant might expect that he will sweat heavily, but might not bother about this. Whether or not this expectancy is then violated might not contribute to the success of exposure. However, we found significant positive correlations between the number of expectancies and pre-treatment anxiety levels, which goes against this explanation^2^.

Our results indicate that the experimental manipulation did not influence treatment effects. Providing verbal instructions that implied that aversive outcomes related to the overt reactions of the audience could not have occurred during VRET did not affect reductions in public speaking anxiety. Notably, most participants rated the instruction that served as manipulation as credible. However, a manipulation check comparing the proportion of testable expectancies about the overt reactions of the audience before and after the experimental manipulation did not reveal the expected decrease in the non-interactive condition. Although it could therefore be questioned whether participants took the information into account, we took several precautions to guarantee this: participants were told to carefully read the information sheet with experimental instructions, to sign it, and the experimenter also orally repeated the gist of the instruction. Notably, these results are in line with findings of a study by Morina et al. (2014). The aim of this study was different from the current study, since they investigated whether the degree of interaction in VR had an effect on the sense of presence participants felt. Participants engaged in free speech dialogues with virtual humans and were assigned to either a condition in which interaction between the individual and the virtual human was possible or to a condition in which such interaction was not possible. Hence, instead of giving verbal information retrospectively, in this study the devices that were used did or did not allow for interaction. Higher levels of presence were reported by the interactive condition. Importantly, there was no effect of the degree of interaction on the reported anxiety levels. This suggests that interaction during VRET was not crucial for eliciting anxiety.

The results of the current study do not provide evidence for expectancy violation as the mechanism driving the effects of VRET. The question then arises which mechanism(s) are at play in VRET. According to Emotional Processing Theory, which has dominated the field for many years, the efficacy of exposure results from the initial activation of fear followed by sustained exposure until fear declines [as originally formulated by Foa and Kozak (1986)]. In particular, it is assumed that fear habituation is crucial for making new incompatible information available and for replacing the activated “fear structure,” consisting of propositions between stimuli, responses and meanings in memory, by a “non-fear structure” (Lang, 1971; Rachman, 1980). In line with the basic assumptions of Emotional Processing Theory, it has been demonstrated that VRET allows for within-session reduction of fear (Wiederhold et al., 2002; Wilhelm et al., 2005; but see Mühlberger et al., 2007). The current study, however, did not include physiological and verbal measures of fear during VRET and as a consequence does not allow for investigating whether habituation in these measures predict treatment effects. This would be an interesting question for future research. However, it should be added that an extensive amount of research shows that habituation is not predictive for the long-term outcome of *in vivo* exposure (e.g., Baker et al., 2010; Culver et al., 2012; Kircanski et al., 2012; Brown et al., 2017).

## Conclusion

In conclusion, our findings confirm a reduction in public speaking anxiety after VRET and suggest that low-cost VR technology could be sufficient for treating anxious individuals. Importantly, the availability of a low-cost device can facilitate the broader dissemination and usage of VRET (Morina et al., 2014). We did not find evidence that participants who were better able to test their feared expectancies benefit more from VRET. Expectancies related to own reactions were evaluated as being more testable in VRET compared to expectancies related to the (overt) reactions of the audience or being negatively evaluated. Our results are not in line with the findings of the fear conditioning study of Raes et al. (2011): the decrease in public speaking anxiety after VRET was not affected by retrospective reasoning about whether or not feared outcomes related to the overt reactions of the audience could occur in VRET. Since this study is a first test of the role of expectancy violation in VRET in a subclinical high-anxious student sample, we opted for a brief exposure intervention. In two sessions of VRET, participants were exposed to public speaking for 27 min in total. It is recommended for future studies to investigate the role of expectancy violation in VRET in clinical samples using more elaborate exposure schemes. Moreover, the sample size of this study was limited and it is recommended to include larger samples in future studies. The exact underlying mechanisms that are at work in VRET are currently not well-known and remain an important topic for further research, especially since this knowledge can drive the further optimization of VRET.

## Data Availability Statement

All datasets generated for this study are included in the article/Supplementary Material.

## Ethics Statement

The studies involving human participants were reviewed and approved by the Ethical Committee of the Faculty of Psychology and Educational Sciences of KU Leuven. The patients/participants provided their written informed consent to participate in this study.

## Author Contributions

SS, YB, TV, and DH contributed to the design of the study. SS collected and analyzed the data. SS and YB wrote the manuscript. TV and DH revised the manuscript on multiple occasions and provided feedback.

## Conflict of Interest

The authors declare that the research was conducted in the absence of any commercial or financial relationships that could be construed as a potential conflict of interest.

## Footnotes

1 Notably, systematic evaluation of the research quality of VRET studies reveals generally low research quality (McCann et al., 2014; Motraghi et al., 2014; Page and Coxon, 2016).

2 PRCS: *r* = 0.63 (*p* < 0.001); BAT_SUDS: *r* = 0.39 (*p* = 0.01); SSPS-N: *r* = 0.62 (*p* < 0.001).
