# Supplementary material for: Virtually Unexpected: No Role for Expectancy Violation in Virtual Reality Exposure for Public Speaking Anxiety
Source: Front Psychol. 2019 Dec 17;10:2849. doi: 10.3389/fpsyg.2019.02849 (PMC6928118; doi:10.3389/fpsyg.2019.02849)
Supplement: Supplementary file 2 [file Table_1.DOCX]

# Supplementary Material

Supplementary Material A:

List of expectancies related to individuals’ own reactions (S), the overt reactions of the audience (A) and negative evaluation (NE).

| 1. I won´t get my facts straight (S) | Yes – No |
| --- | --- |
| 1. They will think that I am incompetent (NE) | Yes – No |
| 1. I will sweat heavily (S) | Yes – No |
| 1. I will have cardiac palpitations (S) | Yes – No |
| 1. They will think that I am not interesting (NE) | Yes – No |
| 1. I will lose control (S) | Yes – No |
| 1. They will think that I am a weird person (NE) | Yes – No |
| 1. I will start blushing (S) | Yes – No |
| 1. People in the audience will ask difficult questions (A) | Yes – No |
| 1. The anxiety will be intolerable (S) | Yes – No |
| 1. I will not be able to cope with it (S) | Yes – No |
| 1. They will think that I am ridiculous (NE) | Yes – No |
| 1. They will think that I am boring (NE) | Yes – No |
| 1. I will freeze (S) | Yes – No |
| 1. I will escape from the situation (S) | Yes – No |
| 1. People in the audience will sigh in reaction to my speech (A) | Yes – No |
| 1. I will suddenly not know what to say (S) | Yes – No |
| 1. They will think that I am weak (NE) | Yes – No |
| 1. I will vomit (S) | Yes – No |
| 1. People in the audience will have a disapproving look on their face in reaction to my speech (A) | Yes – No |
| 1. I will start crying (S) | Yes – No |
| 1. I won’t be able to think (S) | Yes – No |
| 1. People in the audience will yawn in reaction to my speech (A) | Yes – No |
| 1. I will feel dizzy (S) | Yes – No |
| 1. I will hyperventilate (S) | Yes – No |
| 1. People in the audience will make fun of me because of my speech (A) | Yes – No |
| 1. I will be overwhelmed by anxiety and won´t be able to speak anymore (S) | Yes – No |
| 1. People in the audience will criticize me (A) | Yes – No |
| 1. I will feel nauseous (S) | Yes – No |
| 1. They will think that I am unintelligent (NE) | Yes – No |
| 1. I will stutter (S) | Yes – No |
| 1. I will fail (i.e., not succeed in the presentation) (NE) | Yes – No |
| 1. People in the audience will be looking on their mobile phone because they are not interested in my speech (A) | Yes – No |
| 1. I will faint (S) | Yes – No |
| 1. People in the audience will roll their eyes in reaction to my speech (A) | Yes – No |
| 1. People in the audience will frown in reaction to my speech (A) | Yes – No |
| 1. They will think that I am stupid (NE) | Yes – No |
| 1. I will behave hysterically (S) | Yes – No |
| 1. They will think that I am saying stupid things (NE) | Yes – No |
| 1. I will make a bad impression (NE) | Yes – No |
| 1. I will tremble (S) | Yes – No |
| 1. They will think that I look silly (NE) | Yes – No |
| 1. I will panic (S) | Yes – No |
| 1. People in the audience will start talking to each other because they find my speech not interesting (A) | Yes – No |
| 1. I will become crazy (S) | Yes – No |
| 1. They will notice that I am anxious and nervous (NE) | Yes – No |
| 1. I will behave aggressively (S) | Yes – No |
| 1. I will talk in a strange way (S) | Yes – No |
| 1. They will think that I am an idiot/loser (NE) | Yes – No |
| 1. People in the audience will leave in reaction to my speech (A) | Yes – No |

Supplementary material B:

Information sheet that served as the experimental manipulation

**Interactive condition:**

*Below, you can find additional information about the virtual environment in which you practiced public speaking:*

*Despite the fact that it was a virtual audience, the audience was interactive and as a consequence the reactions of the audience were adapted to your presentations. During your presentations, the experimenter could manipulate the reactions of the audience on his PC. This implies that if you, for example, would have said something odd or stupid, you could have noticed this in the reactions (facial expression, laughter, other behavior) of the audience.*

*This technology (including a photorealistic interactive virtual audience) is relatively new within the field of virtual reality and complex to program.*

*Because we aim to further optimize this technology and eventually launch it for commercial use, we ask you to declare that you understood the above information and that you will not communicate about the used technology with other research centers or companies involved in the field of virtual reality.*

**Non-interactive condition:**

*Below, you can find additional information about the virtual environment in which you practiced public speaking:*

*Because it was a virtual audience, the audience was not interactive and as a consequence the reactions of the audience were not adapted to your presentations. The virtual environments consisted of 360 degrees movie clips of an audience that were recorded beforehand and were showed during your speeches. This implies that if you, for example, would have said something odd or stupid, you could not have noticed this in the reactions (facial expression, laughter, other behavior) of the audience.*

*Interactive virtual environments are currently available, but only when avatars are used. In this study, we choose for a photorealistic audience. To date, a photorealistic audience that is able to interact with the user is too complex to program. Therefore we used 360 degrees movie clips of a photorealistic audience that could not interact.*

*Because these movie clips will be further used for commercial purposes, we ask you to declare that you understood the above information and that you will not communicate about the used technology with other research centers or companies involved in the field of virtual reality.*
